# Supplementary material for: Balancing Functional Tradeoffs between Protein Stability and ACE2 Binding in the SARS-CoV-2 Omicron BA.2, BA.2.75 and XBB Lineages: Dynamics-Based Network Models Reveal Epistatic Effects Modulating Compensatory Dynamic and Energetic Changes
Source: Viruses. 2023 May 10;15(5):1143. doi: 10.3390/v15051143 (PMC10221141; doi:10.3390/v15051143)
Supplement: Supplementary file 1 [file viruses-15-01143-s001.zip › viruses-2327705-supplementary.pdf]

Supplementary Materials

Balancing Functional Tradeoffs between Protein Stability and ACE2 Binding in the SARS-CoV-2 Omicron BA.2, BA.2.75 and XBB Lineages: Dynamics-Based Network Models Reveal Epistatic Effects Modulating Compensatory Dynamic and Energetic Changes

Gennady Verkhivker [1,2,*], Mohammed Alshahrani [1] and Grace Gupta [1]

[1] Keck Center for Science and Engineering, Graduate Program in Computational and Data Sciences, Schmid College of Science and Technology, Chapman University, Orange, CA 92866, USA;
alshahrani@chapman.edu (M.A.); grgupta@chapman.edu (G.G.)

[2] Department of Biomedical and Pharmaceutical Sciences, Chapman University School of Pharmacy,
Irvine, CA 92618, USA

* Correspondence: verkhivk@chapman.edu; Tel.: +1-714-516-4586

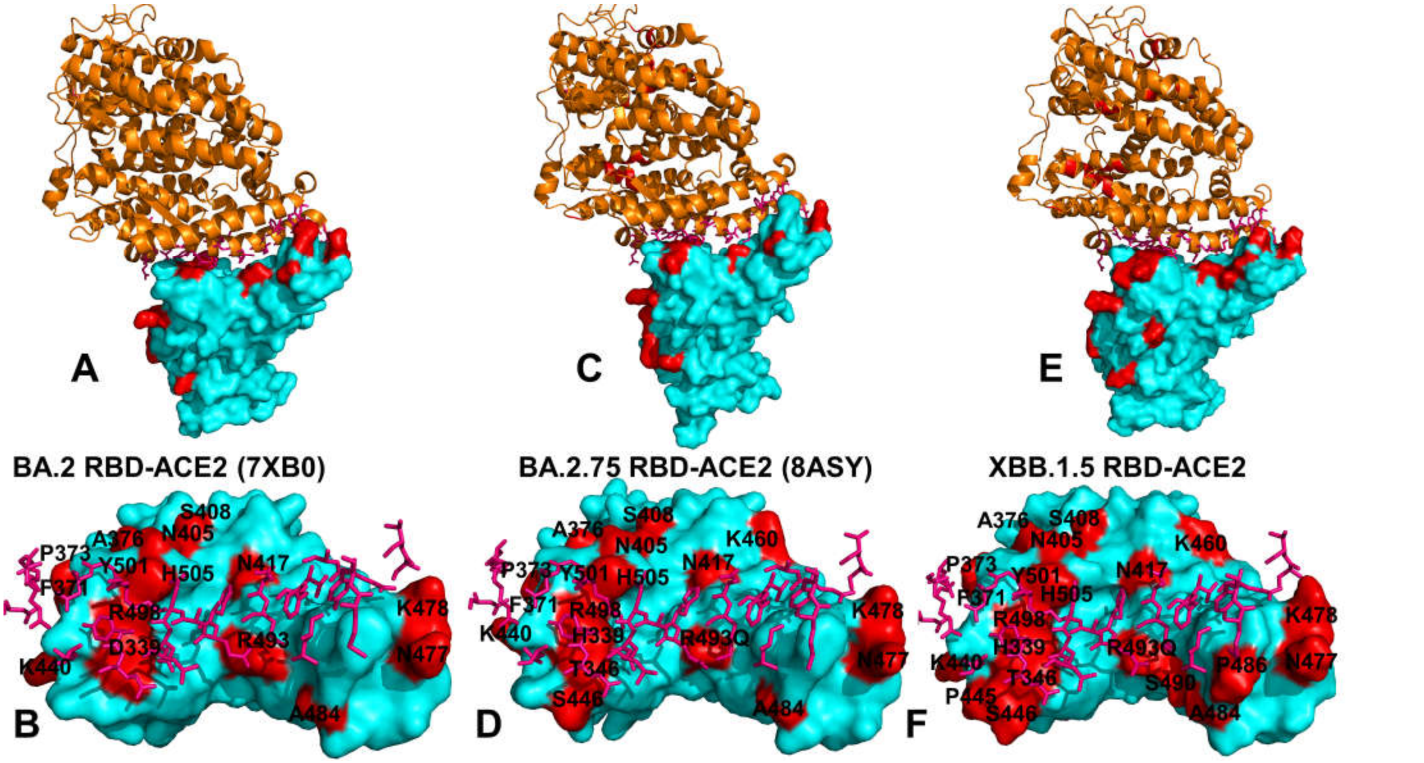

Figure S1. Structural organization of the SARS-CoV-2-RBD Omicron BA.2, BA.2.75, XBB.1 and XBB.1.5 complexes with human ACE2 enzyme. (A) The crystal structure of the Omicron RBD BA.2-ACE2 complex (pdb id 7XB0). The RBD is shown in cyan-colored surface and the bound ACE2 enzyme is in orange ribbons. (B) The RBD-BA.2 is shown in cyan surface from the top view. The ACE2 binding residues are shown in pink sticks. The Omicron RBD BA.2 sites (G339D, S371F, S373P, S375F, T376A, D405N, R408S, K417N, N440K, S477N, T478K, E484A, Q493R, Q498R, N501Y, Y505H) are shown in red surface and annotated. (C) The crystal structure of the Omicron RBD BA.2.75-ACE2 complex (pdb id 8ASY). The RBD is shown in cyan-colored surface and the bound ACE2 enzyme is in orange ribbons. (D) The RBD-BA.2.75 is shown from the top view. The ACE2 binding residues are shown in pink sticks. The Omicron RBD BA.2.75 sites (G339H, S371F, S373P, S375F, T376A, D405N, R408S, K417N, N440K, G446N, N460K, S477N, T478K, E484A, R493Q, Q498R, N501Y, Y505H) are shown in red surface and annotated. (E) The refined structural model of the Omicron RBD XBB.1.5-ACE2 complex. The RBD is shown in cyan-colored surface and the bound ACE2 enzyme is in orange ribbons. (F) The RBD-XBB.1 is shown from the top view and the ACE2 binding residues are shown in pink-colored sticks. The Omicron RBD XBB.1.5 sites (G339H, R346T, L368I, S371F, S373P, S375F, T376A, D405N, R408S, K417N, N440K, V445P, G446S,N460K, S477N, T478K, E484A, F486P, F490S, R493Q, Q498R, N501Y, Y505H) are shown in red-colored surface and annotated.

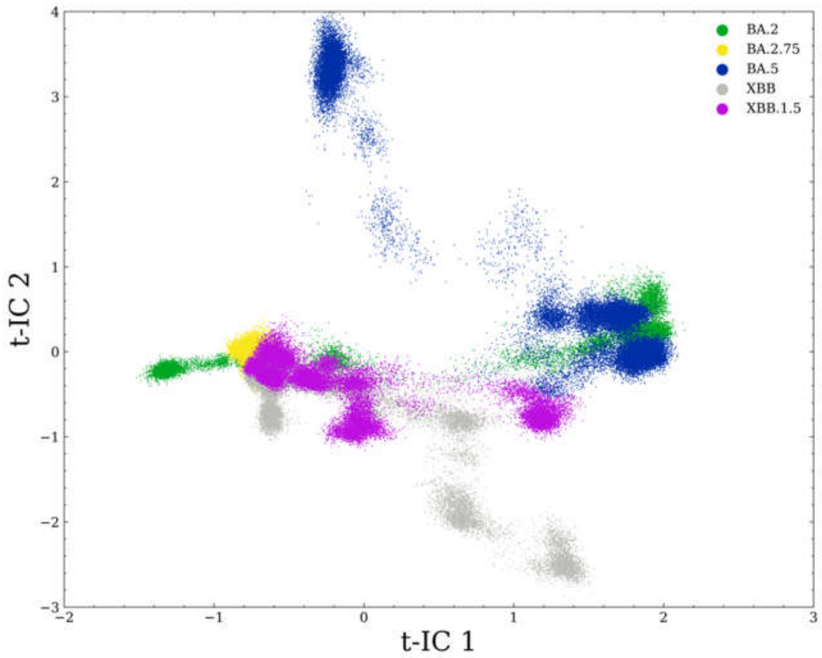

Figure S2. The low-dimensional representation with 2 components (t-IC) obtained from MD simulations of the Omicron RBD-ACE2 complexes. Note that the distribution of BA.2.75 simulation is limited to the yellow area. Other complexes explore much wider areas. To visualize the distribution density of all simulations a hexagon binning is applied. Each hexagon represents a small region, and the color bar shows the frequency of this region.

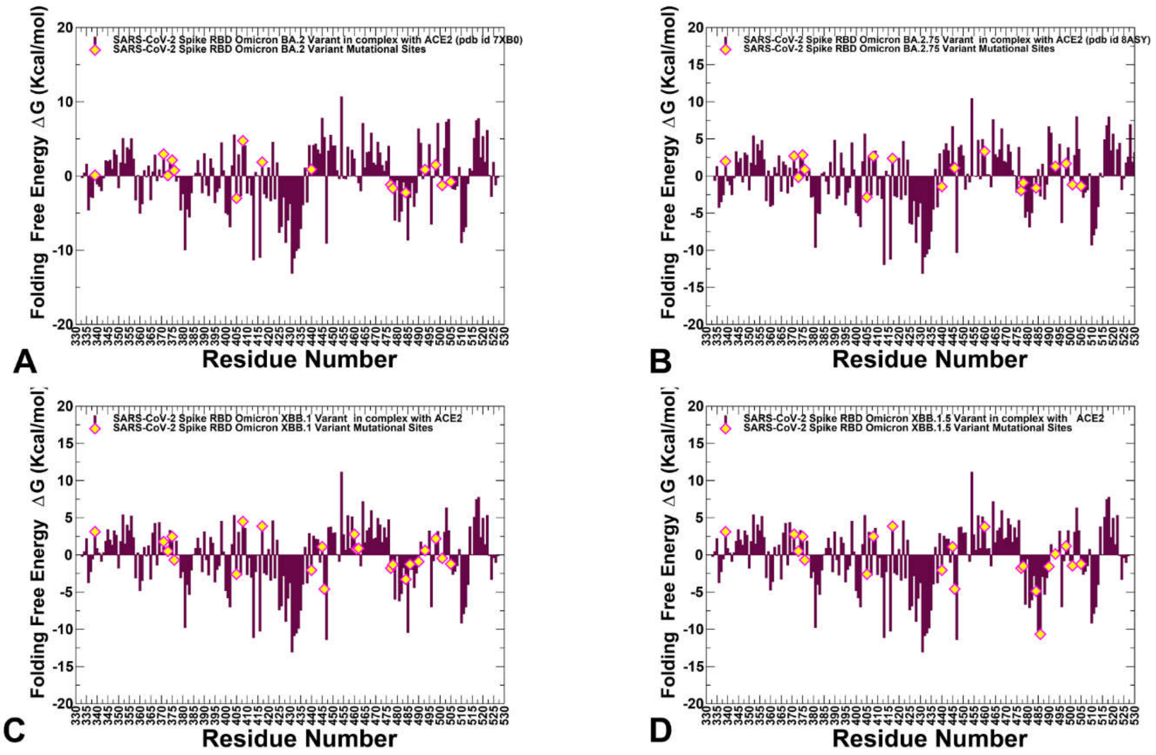

Figure S3. The folding free energies of the RBD residues for the SARS-CoV-2 S Omicron RBD-hACE2 complexes for BA.2, BA.2.75, XBB.1 and XBB.1.5 variants. The folding free energies for the RBD residues in the BA.2 RBD-hACE2 complex (A), BA.2.75 RBD-hACE2 complex (B), XBB.1 RBD-hACE2 complex (C) and XBB.1.5 RBD-hACE2 complex (D). The profiles are shown in maroon-colored bars. The positions of the Omicron mutational sites on the profiles are highlighted in yellow-colored filled diamonds. Note that the XBB.1.5 mutational sites V445P, G446S,N460K, S477N, T478K, E484A, F486P, F490S, R493Q, Q498R, N501Y, Y505H showed favorable stability, particularly evident for F486P mutation.

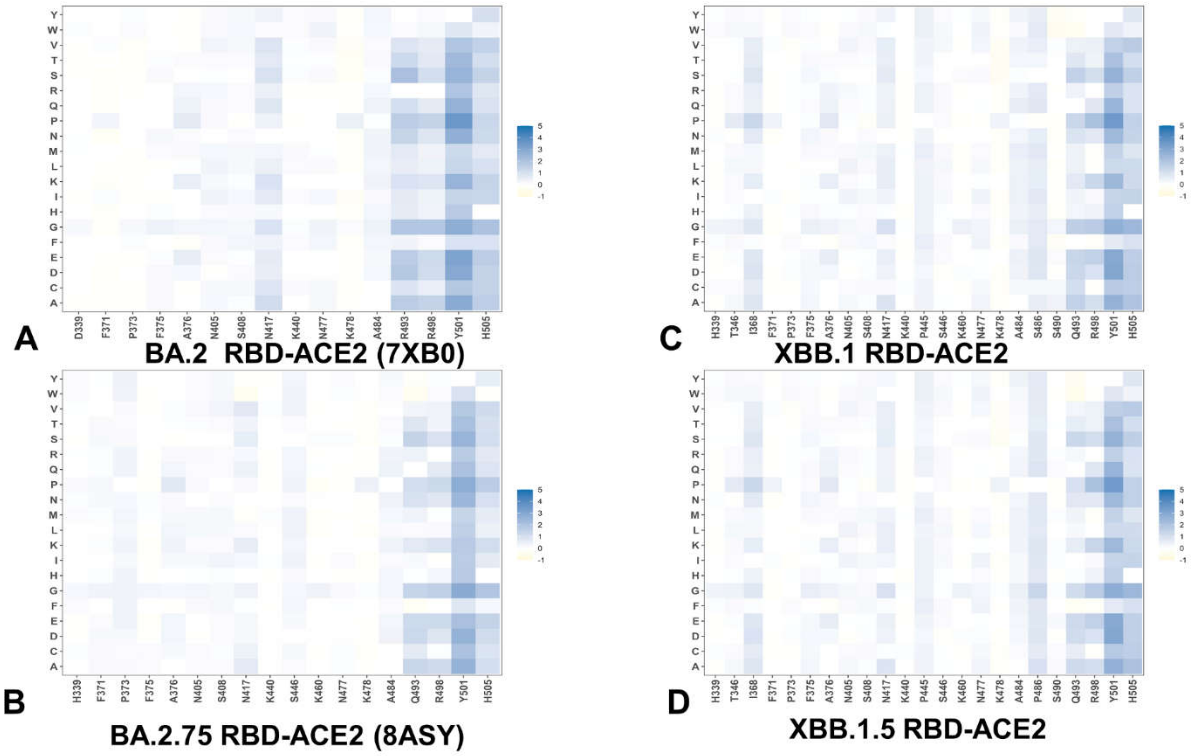

Figure S4. Ensemble-based dynamic mutational profiling of the Omicron RBD mutational sites in the RBD-hACE2 complexes. The mutational scanning heatmaps are shown for the BA.2 mutational sites in the Omicron RBD BA.2-hACE2 complex (A). Mutational scanning heatmap for the BA.2.75 mutational sites in the Omicron RBD BA.2.75-hACE2 complex (B). Mutational heatmap for the XBB.1 mutational sites in the Omicron RBD XBB.1-hACE2 complex (C). Mutational heatmap for the XBB.1.5 mutational sites in the Omicron RBD XBB.1.5-hAC2 (D).

Table S1. The list of the intermolecular contacts in the structure of SARS-CoV-2 BA.2 RBD-ACE2 complex (pdb id 7XB0).

ACE2 Residue	ACE2 Residue Number	ACE2 Chain ID	RBD Residue	RBD Residue Number	RBD Chain ID
ASP	355	A	GLY	502	B
TYR	83	A	ASN	487	B
GLN	24	A	ASN	477	B
ASP	355	A	THR	500	B
ASP	30	A	PHE	456	B
GLN	42	A	TYR	449	B
ASP	38	A	TYR	501	B
GLY	354	A	TYR	501	B
TYR	83	A	TYR	489	B
LEU	45	A	VAL	445	B
LEU	45	A	THR	500	B
LYS	353	A	TYR	495	B
GLN	24	A	PHE	486	B
HIS	34	A	LEU	455	B
LYS	353	A	GLY	502	B
LYS	353	A	THR	500	B
SER	19	A	ALA	475	B
LYS	31	A	LEU	455	B
LEU	79	A	PHE	486	B
GLN	24	A	TYR	489	B
TYR	41	A	ARG	498	B
ASN	330	A	THR	500	B
THR	20	A	ALA	475	B

LYS	353	A	VAL	503	B
ARG	357	A	THR	500	B
LYS	353	A	HIS	505	B
TYR	41	A	TYR	501	B
TYR	83	A	PHE	486	B
GLU	35	A	ARG	493	B
ASP	38	A	ARG	498	B
LEU	79	A	GLY	485	B
GLY	354	A	GLY	502	B
GLN	24	A	ALA	475	B
GLY	354	A	THR	500	B
ASP	38	A	GLY	496	B
PHE	28	A	TYR	489	B
ALA	25	A	ASN	487	B
THR	27	A	TYR	489	B
THR	27	A	PHE	456	B
SER	19	A	GLY	476	B
HIS	34	A	TYR	453	B
GLY	354	A	VAL	503	B
ASP	30	A	ASN	417	B
MET	82	A	PHE	486	B
GLU	37	A	HIS	505	B
GLY	354	A	HIS	505	B
LEU	45	A	ARG	498	B
ASP	38	A	TYR	449	B
THR	27	A	ALA	475	B

ASP	355	A	TYR	501	B
LYS	353	A	ARG	403	B
HIS	34	A	ARG	493	B
GLN	325	A	GLN	506	B
TYR	41	A	THR	500	B
HIS	34	A	ARG	403	B
GLN	24	A	GLY	476	B
GLY	352	A	GLY	502	B
GLN	24	A	ASN	487	B
GLN	42	A	ARG	498	B
LYS	31	A	ARG	493	B
ASP	38	A	TYR	495	B
SER	19	A	ASN	477	B
LYS	353	A	TYR	501	B
ASP	30	A	LEU	455	B
LYS	31	A	TYR	489	B
LYS	31	A	PHE	456	B
LYS	31	A	GLY	485	B
HIS	34	A	ASN	417	B
THR	27	A	TYR	473	B
THR	20	A	ASN	477	B
GLY	326	A	THR	500	B
GLN	42	A	GLY	446	B
GLY	352	A	TYR	501	B
ASN	330	A	PRO	499	B

Table S2. The list of the intermolecular contacts in the structure of SARS-CoV-2 BA.2.75 RBD-ACE2 complex (pdb id 8ASY).

ACE2 Residue	ACE2 Residue Number	ACE2 Chain ID	RBD Residue	RBD Residue Number	RBD Chain ID
ARG	357	A	THR	500	B
LYS	31	A	PHE	456	B
GLU	35	A	GLN	493	B
ASP	38	A	TYR	449	B
ASP	355	A	GLY	502	B
LYS	353	A	GLY	496	B
GLU	37	A	HIS	505	B
LEU	45	A	THR	500	B
THR	27	A	ALA	475	B
ASN	330	A	THR	500	B
TYR	83	A	TYR	489	B
ASP	30	A	PHE	456	B
THR	27	A	TYR	489	B
ASP	355	A	THR	500	B
LYS	353	A	TYR	495	B
HIS	34	A	GLN	493	B
PHE	28	A	TYR	489	B
PHE	28	A	ASN	487	B
ASP	38	A	GLY	496	B
SER	19	A	ASN	477	B
LYS	353	A	HIS	505	B
ASP	38	A	TYR	495	B

LYS	353	A	GLY	502	B
SER	19	A	ALA	475	B
THR	27	A	PHE	456	B
GLY	354	A	TYR	501	B
GLN	24	A	ASN	477	B
LEU	79	A	PHE	486	B
GLN	42	A	ARG	498	B
TYR	83	A	ASN	487	B
LYS	353	A	TYR	501	B
LYS	31	A	LEU	455	B
GLY	354	A	THR	500	B
LYS	353	A	THR	500	B
TYR	83	A	PHE	486	B
GLN	24	A	TYR	489	B
LEU	45	A	VAL	445	B
ASP	30	A	LEU	455	B
LYS	353	A	ARG	498	B
HIS	34	A	ASN	417	B
ASP	38	A	TYR	501	B
GLY	354	A	HIS	505	B
GLY	354	A	GLY	502	B
GLN	42	A	TYR	449	B
TYR	41	A	TYR	501	B
MET	82	A	PHE	486	B
HIS	34	A	TYR	453	B
GLY	352	A	TYR	501	B

ALA	25	A	ASN	487	B
TYR	41	A	THR	500	B
SER	19	A	GLY	476	B
LYS	31	A	TYR	489	B
LYS	31	A	GLN	493	B
GLY	354	A	VAL	503	B
ASP	355	A	TYR	501	B
ASP	38	A	ARG	498	B
TYR	41	A	ARG	498	B
LEU	45	A	ARG	498	B
GLN	24	A	ALA	475	B
GLN	24	A	GLY	476	B
GLN	42	A	SER	446	B
HIS	34	A	LEU	455	B
THR	27	A	TYR	473	B
GLN	24	A	ASN	487	B
LYS	31	A	PHE	490	B
GLN	325	A	VAL	503	B
GLN	24	A	PHE	486	B

Table S3. The list of the intermolecular contacts in the structure of SARS-CoV-2 XBB.1 RBD-ACE2 complex (structural model).

ACE2 Residue	ACE2 Residue Number	ACE2 Chain ID	RBD Residue	RBD Residue Number	RBD Chain ID
LEU	79	A	GLY	485	B
ALA	25	A	ASN	487	B
HIS	34	A	ARG	403	B
LEU	45	A	THR	500	B
GLN	24	A	ALA	475	B
TYR	83	A	ASN	487	B
ASN	330	A	THR	500	B
ASP	30	A	ASN	417	B
ASP	38	A	SER	494	B
TYR	41	A	ARG	498	B
GLN	42	A	ARG	498	B
TYR	83	A	TYR	489	B
GLY	354	A	GLY	502	B
LYS	31	A	SER	490	B
HIS	34	A	TYR	453	B
LYS	353	A	GLY	504	B
TYR	41	A	THR	500	B
THR	20	A	ASN	477	B
LEU	79	A	SER	486	B
THR	27	A	ALA	475	B
LYS	353	A	GLY	502	B
ALA	36	A	ARG	493	B
MET	82	A	ASN	487	B

HIS	34	A	ASN	417	B
GLY	326	A	THR	500	B
GLN	24	A	TYR	489	B
ASP	38	A	TYR	495	B
GLY	354	A	TYR	501	B
LYS	31	A	TYR	489	B
GLY	354	A	HIS	505	B
GLN	24	A	GLY	476	B
PHE	28	A	TYR	489	B
ASP	355	A	GLY	502	B
TYR	83	A	SER	486	B
ASP	38	A	ARG	498	B
ASN	330	A	PRO	499	B
GLN	42	A	TYR	449	B
LYS	353	A	TYR	501	B
HIS	34	A	SER	494	B
LEU	45	A	PRO	445	B
LYS	353	A	HIS	505	B
LYS	31	A	LEU	455	B
LYS	353	A	VAL	503	B
GLN	24	A	ASN	477	B
ASP	38	A	TYR	501	B
LEU	45	A	ARG	498	B
ASP	355	A	TYR	501	B
LYS	31	A	ALA	484	B
LEU	39	A	ARG	493	B

LYS	353	A	TYR	495	B
HIS	34	A	ARG	493	B
MET	82	A	SER	486	B
LYS	353	A	GLN	506	B
LYS	31	A	PHE	456	B
GLY	352	A	TYR	501	B
ASP	30	A	LEU	455	B
LYS	31	A	CYS	488	B
GLY	354	A	THR	500	B
SER	19	A	GLY	476	B
GLN	325	A	GLN	506	B
PHE	28	A	PHE	456	B
ASP	38	A	TYR	449	B
HIS	34	A	GLU	406	B
ASP	30	A	PHE	456	B
THR	27	A	TYR	473	B
TYR	41	A	TYR	501	B
GLN	42	A	SER	446	B
THR	27	A	PHE	456	B
SER	19	A	ASN	477	B
GLN	24	A	ASN	487	B
ASP	38	A	ARG	493	B
ARG	357	A	THR	500	B
HIS	34	A	LEU	455	B
GLU	35	A	ARG	493	B
GLU	37	A	HIS	505	B

THR	27	A	TYR	489	B
ASP	355	A	THR	500	B
SER	19	A	ALA	475	B
ASP	38	A	GLY	496	B
GLY	354	A	VAL	503	B

Table S4. The list of the intermolecular contacts in the structure of SARS-CoV-2 XBB.1.5 RBD-ACE2 complex (structural model).

ACE2 Residue	ACE2 Residue Number	ACE2 Chain ID	RBD Residue	RBD Residue Number	RBD Chain ID
GLU	37	A	HIS	505	B
GLN	42	A	TYR	449	B
LEU	45	A	PRO	445	B
TYR	41	A	ARG	498	B
SER	19	A	ALA	475	B
ASP	38	A	TYR	501	B
GLY	354	A	GLY	502	B
GLN	24	A	ASN	477	B
PHE	28	A	TYR	489	B
TYR	41	A	THR	500	B
HIS	34	A	ARG	403	B
GLY	354	A	THR	500	B
LEU	39	A	ARG	493	B
GLN	24	A	ALA	475	B
GLN	325	A	GLN	506	B
TYR	83	A	ASN	487	B
ASP	30	A	LEU	455	B
GLY	326	A	THR	500	B
MET	82	A	ASN	487	B
TYR	83	A	TYR	489	B
THR	20	A	ASN	477	B
LYS	31	A	PHE	456	B
LYS	353	A	GLY	504	B

LYS	31	A	ARG	493	B
ASP	38	A	ARG	493	B
ASP	38	A	ARG	498	B
ASP	30	A	PHE	456	B
HIS	34	A	ASN	417	B
LYS	31	A	TYR	489	B
GLN	24	A	ASN	487	B
SER	19	A	GLY	476	B
ARG	357	A	THR	500	B
ASN	330	A	PRO	499	B
GLN	24	A	TYR	489	B
THR	27	A	TYR	489	B
HIS	34	A	LEU	455	B
HIS	34	A	SER	494	B
SER	19	A	ASN	477	B
ASP	355	A	TYR	501	B
TYR	83	A	PRO	486	B
LYS	31	A	LEU	455	B
LYS	353	A	TYR	501	B
MET	82	A	PRO	486	B
LEU	79	A	PRO	486	B
ASP	38	A	SER	494	B
GLY	352	A	TYR	501	B
THR	27	A	ALA	475	B
LYS	353	A	HIS	505	B
HIS	34	A	TYR	453	B

ASN	330	A	THR	500	B
LYS	353	A	VAL	503	B
ASP	38	A	TYR	449	B
ASP	30	A	ASN	417	B
LEU	45	A	ARG	498	B
HIS	34	A	ARG	493	B
GLN	42	A	ARG	498	B
LEU	45	A	THR	500	B
LYS	353	A	TYR	495	B
THR	27	A	TYR	473	B
TYR	41	A	TYR	501	B
LYS	353	A	GLN	506	B
THR	27	A	PHE	456	B
ASP	355	A	GLY	502	B
ALA	36	A	ARG	493	B
GLN	42	A	SER	446	B
ASP	38	A	TYR	495	B
LYS	353	A	GLY	502	B
GLY	354	A	TYR	501	B
GLU	35	A	ARG	493	B
HIS	34	A	GLU	406	B
ASP	355	A	THR	500	B
GLY	354	A	HIS	505	B
ASP	38	A	GLY	496	B
GLY	354	A	VAL	503	B
LEU	79	A	GLY	485	B

GLN	24	A	GLY	476	B
